# An Interesting Case

**Published:** 1889-03

**Authors:** 


### AN INTERESTING CASE.

A correspondent cites an unusual case of non-eruption of permanent teeth. The subject—a young lady of nineteen—has never erupted either of the four second bicuspids, the left superior lateral, nor the left superior cuspid. The deciduous teeth were models in form, position, and texture, and were shed at the usual time, with normal absorption of roots, with the exception of the left superior cuspid, which was removed with forceps on the first symptom of loosening, the right cuspid being already fully erupted and the left apparently ready to make its appearance.

There is no space for the bicuspids. A plate has been worn for some years, both to fill the unsightly gap, and with the hope that the irritation produced by the pressure of the artificial teeth might hasten the growth of the latent germs of the lateral and cuspid. A tooth is now erupting posterior to the right inferior second molar. Whether it will prove to be a missing second bicuspid, or a third molar, remains to be seen, only one cusp being as yet partially visible which has retreated several times beneath the overhanging swollen gum.

